# All‐inside meniscal repair using polyether ether ketone versus all‐suture anchors: No difference in failure rates at three‐follow‐up in a retrospective cohort of 2253 patients

**DOI:** 10.1002/ksa.70026

**Published:** 2025-09-16

**Authors:** Christoffer von Essen, Riccardo Cristiani, Alexander Sandon, Anders Stålman

**Affiliations:** ^1^ Department of Molecular Medicine and Surgery Section of Sports Medicine, Karolinska Institutet Stockholm Sweden; ^2^ Stockholm Sports Trauma Research Center (SSTRC), FIFA Medical Centre of Excellence Stockholm Sweden

**Keywords:** all‐inside, all‐suture, failure, meniscectomy, meniscus, repair

## Abstract

**Purpose:**

To compare mid‐term failure rates of next‐generation all‐inside meniscal repair using polyether ether ketone (PEEK) anchors versus all‐suture anchors in a large patient cohort. The null hypothesis was that there would be no difference in failure rates between PEEK and all‐suture anchors.

**Methods:**

A retrospective cohort study was conducted on all consecutive patients who underwent arthroscopic all‐inside meniscal repair between January 2015 and June 2022 at Capio Artro Clinic, Stockholm, Sweden. Patients were included if they had isolated or anterior cruciate ligament reconstruction (ACLR) associated medial or lateral meniscal repairs using either PEEK or all‐suture devices. Revision meniscal repairs were excluded to ensure a homogeneous cohort. The primary outcome was failure of meniscal repair, defined as reoperation with partial or total meniscectomy or re‐suture within 3 years. Logistic regression was used to identify predictors of failure. Between‐group comparisons were performed using chi‐square and *t*‐tests.

**Results:**

Of the total cohort of 2253 patients, 271 patients received all‐suture anchors and 1982 received PEEK anchors. The overall reoperation rate was 17.3% (47/271) in the all‐suture group and 20.7% (410/1982) in the PEEK group, with no statistically significant difference. Subgroup analysis by laterality (medial [21.5%, 28/271] vs. lateral meniscus repair [27.1%, 324/1982]), or isolated repair versus ACLR with repair did not reveal significant differences in failure rates for the different devices. Logistic regression further revealed that isolated meniscal repair (odds ratio [OR]: 3.01; 95% confidence Interval [CI]: 2.41–3.76; *p* < 0.001), female gender (OR: 1.30, 95% CI: 1.05–1.61; *p* = 0.017), and medial meniscus repair (OR: 2.89; 95% CI: 2.27–3.69; *p* < 0.001) were significant predictors of meniscal repair failure.

**Conclusion:**

All‐suture and PEEK‐based all‐inside meniscal repair devices demonstrated comparable failure rates within 3 years. Device type was not associated with increased risk of failure, whereas isolated repair, medial meniscal repair, and female gender were significant predictors. These findings support the clinical effectiveness of both anchor types, allowing device selection to be based on surgeon preference and tear characteristics rather than concerns over differential failure risk.

**Level of Evidence:**

Level III retrospective cohort study.

**Abbreviations**
ACL: anterior cruciate ligament
ACLR: anterior cruciate ligament reconstruction
CI: confidence Interval
MRI: magnetic resonance imaging
OR: odds ratio
PEEK: polyether ether ketone
SD: standard deviation
SE: standard error

## INTRODUCTION

There are many techniques for meniscus repair, including inside‐out, outside in and all‐inside. While the inside‐out technique has been considered the gold standard, the development of all‐inside devices has introduced many advantages such as, reduced surgical morbidity, shorter operative times, and simpler technical application [22], leading to a paradigm shift [1]. These devices secure the anchor to the capsule peripheral to the meniscus and prove reliable in terms of failure rates [11, 20].

While many modern all‐inside devices utilise polyether ether ketone (PEEK)‐based anchors, which have demonstrated to have similar biomechanical properties and clinical healing to inside‐out repairs [6, 7, 22], some manufacturers replaced the rigid anchor with all‐suture anchors. All‐suture anchors utilise a similar technology that has been utilised in shoulder surgery for rotator‐cuff repairs and instability with good biomechanical results [9, 14]. These all‐suture anchors also deploy posterior to the capsule and expand when the repair is tensioned [9].

Biomechanical studies suggest that all‐suture anchors may provide higher load to failure and reduced gapping compared to PEEK anchors [2]. However, there is a lack of clinical studies comparing failure rates between all‐suture anchors and PEEK‐anchors.

This study aimed to investigate the difference in mid‐term failure rates between next‐generation all‐inside meniscus repairs using PEEK anchors compared to all‐suture anchors. Although biomechanical studies have demonstrated that all‐suture anchors may exhibit higher ultimate load to failure and reduced gapping compared to PEEK‐based anchors, the clinical relevance of these findings remains uncertain. These biomechanical advantages do not necessarily translate into superior healing or lower failure rates in vivo, where biological and patient‐related factors play a significant role. Therefore, the null hypothesis of this study was that there would be no significant difference in mid‐term failure rates between meniscal repairs performed with PEEK anchors and those performed with all‐suture anchors.

## METHODS

Ethical approval for this study was obtained from the regional ethics committee (Karolinska Institutet, DNR 2024‐06048‐01). The Strengthening the Reporting of Observational Studies in Epidemiology (STROBE) statement was used as guidance to report this study to increase scientific quality.

Patients who underwent arthroscopic all‐inside meniscal repair from January 2015 to June 2022 at Capio Artro Clinic, Stockholm, Sweden, were identified retrospectively using procedural (ICD‐10) codes, m232, S832 and S837. Electronic medical records and operative reports were reviewed, and patient characteristics were collected.

Inclusion criteria were: (1) medial or lateral all‐inside meniscal repair, (2) isolated meniscal repair or meniscal repair in combination with anterior cruciate ligament (ACL) reconstruction (ACLR) and (3) no previous meniscal surgery in the index knee. Patients were excluded if the meniscal repair was concomitant with a multiligamentous injury. All repairs with an additional inside‐out or outside‐in repair technique were excluded, as well as isolated meniscal root repairs. If the repair was done with a combination of all‐suture and PEEK‐devices, it was also excluded. Re‐repairs were excluded to ensure homogeneity and avoid confounding from prior surgical interventions. The primary study endpoint was the failure of meniscal repair. Failure was defined as reoperation with a partial or total meniscal resection within 3 years. Failures were identified through electronic medical records using procedure codes for partial or total meniscectomy. Patients revised at other centres may not have been captured. Previous studies have shown that most meniscal repair failures occur within the first two years postoperatively, with diminishing rates thereafter [13, 21]. This study expanded the follow‐up to 3 years, to also address intermediate‐term failures.

### Surgical technique

All surgical procedures were performed by fellowship‐trained surgeons, in total 22 different surgeons. Standard arthroscopic portals were utilised. After a diagnostic arthroscopy, the tear underwent preparation in the form of rasping the tear site and adjacent synovium, followed by anatomic reduction. The repair was performed with an all‐inside technique either with a PEEK‐device (FasT‐fix360, Smith & Nephew) or an all‐suture‐device (FiberStitch). Suture configuration (vertical, horizontal or oblique) and the number of sutures were determined by the surgeon based on tear morphology and stability. ACLRs were performed with a single‐bundle technique with either a hamstring, bone‐patellar tendon‐bone, or quadriceps tendon autografts.

All patients adhered to a standardised rehabilitation protocol, using a hinged brace for a total of 6 weeks, with an increase of 30° of flexion every 2 weeks (0°–30° 1st and 2nd week, 0°–60° 3rd and 4th, and 0°–90° 5th and 6th weeks), with full weight bearing allowed immediately after surgery, except for radial tears where weight‐bearing was restricted for 6 weeks. Return to pivoting sports was not recommended before 4 months for isolated meniscal repairs and 9 months for combined ACLR procedures.

### Statistical analysis

Descriptive statistics were reported as mean ± standard deviation (SD) for continuous variables and as frequencies and percentages for categorical variables. Normality of continuous variables was assessed using the Shapiro–Wilk test. Parameters analysed included age, gender, meniscal laterality, ACLR status, anchor type, and reoperation status. Between‐group comparisons were performed using independent‐samples Student's *t*‐test for continuous variables and Pearson's chi‐square test for categorical variables. Logistic regression analysis was conducted to identify predictors of failure, with device type, gender, meniscal laterality (medial vs. lateral), repair type (isolated vs. concomitant ACLR), and age (dichotomised into classes close to the median [≥30 vs. <30 years]) as independent variables and reoperation as dependent variable. Results were reported as odds ratios (OR) with 95% confidence intervals (CIs). A *p*‐value < 0.05 was considered statistically significant. All analyses were performed using SPSS version 25.0 (IBM Corp.).

## RESULTS

A total of 2876 patients were initially reviewed. After applying inclusion and exclusion criteria, 2253 patients were included in the study, of whom 271 (12.0%) underwent meniscal repair using all‐suture anchors and 1982 (88.0%) using PEEK anchors. The mean age was 27.0 ± 11.0 years, and 56.0% were male. Baseline characteristics were comparable between groups, except for a higher proportion of medial meniscal repairs in the PEEK group (60.4% vs. 47.6%, *p* < 0.01). Detailed patient demographics are presented in Table 1.

**Table 1:** Patient characteristics.

	All‐suture	PEEK	*p* value
No. patients	271	1982	
Age, years (SD)	27.6 (±10.5)	26.3 (±10.5)	
Gender			
Male	150 (55.4)	1111 (56.1)	0.83
Meniscus			<0.01
Medial	129 (47.6)	1197 (60.4)	
Lateral	142 (52.4)	785 (39.6)	
ACL			
Concomitant ACLR	140 (51.7)	1111 (56.1)	0.17

*Note*: Data are reported as *n* (%) unless otherwise indicated.

Abbreviations: ACL, anterior cruciate ligament; ACLR, anterior cruciate ligament reconstruction; No, number of; PEEK, polyether ether ketone; SD, standard deviation.

The overall failure rate, defined as reoperation with partial or total meniscectomy within 3 years, was 20.3% (457/2,253). The failure rate was 17.3% (47/271) in the all‐suture group and 20.7% (410/1,982) in the PEEK group, with no statistically significant difference. Subgroup analysis revealed no significant differences in failure rates between anchor types when stratified by meniscal laterality and concomitant ACLR (Table 2).

**Table 2:** Failure rates of meniscal repair in study groups and depending by laterality and concomitant ACLR.

	All‐suture	PEEK	*p* value
No. patients	271	1982	
Reoperation	47 (17.3)	410 (20.7)	0.19
Medial meniscal repair	28 (21.7)	324 (27.1)	0.19
Lateral meniscal repair	19 (13.4)	86 (11.0)	0.41
Concomitant ACLR	17 (36.2)	144 (35.1)	0.79

*Note*: Data are reported as *n* (%) unless otherwise indicated.

Abbreviations: ACLR, anterior cruciate ligament reconstruction; No., number of; PEEK, polyether ether ketone.

The logistic regression analysis revealed that meniscal repair without ACLR (OR: 3.01; 95% CI: 2.41–3.76; *p* < 0.001), medial meniscal repair (OR: 2.89; 95% CI: 2.27–3.69; *p* < 0.001) and female gender (OR: 1.30; 95% CI: 1.05–1.61; *p* = 0.017) increased the odds for failure within 3 years. No significant relationship was found between type of all‐inside device or age (Table 3).

**Table 3:** Factors affecting failure within 3 years.

	B regression coefficient	SE	OR (95% CI)	*p* value
All‐inside suture	0.18	0.18	1.19 (0.82–1.69)	0.34
Medial meniscus repair	1.06	0.12	2.89 (2.27–3.69)	<0.001
Isolated meniscus repair (no concomitant ACLR)	1.10	0.11	3.01 (2.41–3.76)	<0.001
Female gender	0.26	0.11	1.30 (1.05–1.61)	0.02
Age >30	0.21	0.12	1.02 (0.81–1.28)	0.87

Abbreviations: ACLR, anterior cruciate ligament reconstruction; CI, confidence interval; OR, odds ratio; PEEK, polyether ether ketone; SE, standard error.

Model fit was assessed using the Hosmer‐Lemeshow test (*p* = 0.42), indicating good calibration. The Nagelkerke pseudo *R*² was 0.18, suggesting moderate explanatory power. Multicollinearity was evaluated using variance inflation factors (VIF), and all predictors had VIF < 2, indicating no significant multicollinearity.

## DISCUSSION

The primary finding of this study was that next‐generation all‐inside PEEK and all‐suture anchors demonstrated comparable mid‐term failure rates following meniscal repair. These findings support the null hypothesis, as no statistically significant difference in failure rates was observed between PEEK and all‐suture anchors. In a large cohort of over 2200 patients, no significant difference in reoperation rates was observed between anchor types at 3‐year follow‐up. Instead, isolated meniscal repair, medial meniscal repair, and female gender were identified as independent predictors of failure. These results support the clinical equivalence of both anchor systems and highlight patient‐ and tear‐specific factors as more critical determinants of repair success.

The overall reoperation rate observed in this study is consistent with previously published data [11, 19, 20].

Biomechanical studies have shown that all‐inside meniscal repair devices have evolved significantly, with both PEEK and all‐suture anchors demonstrating adequate mechanical performance. PEEK‐based devices have been reported to provide biomechanical and clinical outcomes comparable to traditional inside‐out techniques [22]. However, comparative studies by Barber et al. and Bachmaier et al. have demonstrated that next‐generation all‐suture all‐inside devices exhibit significantly higher ultimate load to failure than PEEK anchors [2, 3]. Despite this mechanical advantage, the present study found no corresponding improvement in clinical outcomes with all‐suture anchors.

This discrepancy may be explained by the fact that ultimate failure load is not the sole determinant of clinical success. Displacement, or ‘gapping’, defined as the separation between the edges of the repaired meniscus under load, may be a more relevant biomechanical parameter. Experimental studies have shown that displacement in PEEK repairs ranges from 1.4 to 11 mm [3, 19], and no significant difference in displacement has been observed between PEEK and all‐suture devices in cadaveric models [10, 15]. Furthermore, in vivo, meniscal tears are more likely to experience compressive and shear forces rather than distraction during knee motion [16, 17]. These forces are not fully replicated in cadaveric testing, which may limit the translational relevance of biomechanical superiority observed in laboratory settings.

The biomechanical equivalence in displacement and the predominance of shear forces in vivo may help explain the similar failure rates observed between anchor types in this study. These findings underscore the importance of considering both mechanical and biological factors when evaluating meniscal repair outcomes.

Medial meniscal repairs exhibited significantly higher failure rates than lateral repairs, consistent with prior literature [12, 20]. This may be attributed to the biomechanical demands placed on the medial meniscus, which bears greater load and has more limited vascular supply [12]. These anatomical and functional differences likely contribute to the increased susceptibility to re‐tear and impaired healing.

Patients undergoing isolated meniscal repair had a threefold increased risk of failure compared to those with concomitant ACLR. This finding supports the hypothesis that the biological environment during ACLR—characterised by increased vascularity and growth factor release—may enhance meniscal healing. This is consistent with prior studies reporting improved outcomes in combined procedures [4, 5, 8, 20].

Female gender was associated with a modest but statistically significant increased risk of failure. While the underlying mechanisms remain unclear, potential contributors include hormonal influences, anatomical differences, and variations in activity levels. Schweizer et al. [20] conducted a meta‐analysis and identified female gender as a risk factor for meniscal failure. Similar, a large dual‐centre study, Rönnblad et al. [18]. also reported that female gender was associated with a higher risk of failure. In contrast, Ow et al. [13] conducted a systematic review and did not find female gender to be a consistent predictor for failure. Further research is warranted to clarify this association.

Failure is generally defined as the need for subsequent partial or total meniscectomy [18, 20], a definition adopted in the present study as well. However, this definition does not distinguish whether the reoperation addressed the original tear site or a new lesion, nor does it account for partial healing. Magnetic resonance imaging (MRI) may serve as a tool for assessing meniscal healing, although interpretation can be challenging due to persistent signal changes that may not correlate with clinical symptoms [23]. Additionally, detailed characterisation of tear morphology, including size, chronicity, location, and vascular zone, was not available in this study but may significantly influence healing potential and failure risk. A more detailed investigation into these aspects could contribute to a better understanding of the underlying mechanisms leading to meniscal re‐tears. Research has indicated that the primary failure mechanism of PEEK devices is suture breakage or rupture, wherein the suture may fail under lower loads before cutting through the meniscus [10]. In contrast, all‐suture devices predominantly fail due to meniscal cutout, highlighting differences in the failure patterns of these fixation methods [10]. These distinctions may have implications for anchor selection in specific tear patterns. Future studies should include imaging follow‐up and detailed intraoperative characterisation of tear morphology (e.g., length, chronicity, vascular zone) to better predict outcomes and guide implant selection.

The findings in this study have practical implications for surgical decision‐making. Given the comparable failure rates between anchor types, device selection may be guided by surgeon experience, technical preference, and tear morphology.

The principal strength of this study lies in its analysis of a large patient cohort, supported by a meticulous and comprehensive review of medical records to ensure accurate identification of all reoperations. Furthermore, all surgical procedures were performed at a high‐volume, specialised centre for arthroscopic knee surgery, which enhances the internal validity of the findings. However, this may also limit the generalisability of the results to other clinical settings with differing levels of surgical expertise or patient populations.

The present study has several limitations that should be acknowledged. First, the retrospective design inherently introduces potential sources of bias. The reliance on existing medical records may have limited the completeness and accuracy of the data, particularly in capturing all relevant clinical variables and outcomes. Second, the absence of postoperative imaging, such as MRI, restricts the ability to assess meniscal healing objectively. Without imaging follow‐up, it is challenging to differentiate between symptomatic and asymptomatic failures or to evaluate the extent of partial healing, which may have influenced the interpretation of reoperation rates. Third, the study did not account for detailed tear characteristics, including size, location, morphology, and chronicity. These factors are known to significantly impact healing potential and failure risk, and their omission may confound the comparison between PEEK and all‐suture anchor devices. Fourth, the selection of repair devices was based on surgeon preference. This introduces variability related to individual surgeon experience, technical approach, and perceived complexity of the tear, which may have affected the choice of device and subsequent outcomes. Finally, there is no data if the patients underwent meniscal resection at another clinic.

## CONCLUSION

All‐suture and PEEK‐based all‐inside meniscal repair devices demonstrated comparable failure rates within 3 years. Device type was not associated with increased risk of failure, whereas isolated repair, medial meniscal repair and female gender were significant predictors. These findings support the clinical effectiveness of both anchor types, allowing device selection to be based on surgeon preference and tear characteristics rather than concerns over differential failure risk.

## AUTHOR CONTRIBUTIONS

**Christoffer von Essen**: Conceptualisation; data processing; statistical analysis; methodology; manuscript writing. All authors: Methodology; critical review of the manuscript. All authors commented on previous versions of the manuscript. All authors read and approved the final manuscript.

## CONFLICT OF INTEREST STATEMENT

The authors declare no conflicts of interest.

## ETHICS STATEMENT

Ethical permission for this study was obtained from the Regional Ethics Committee of Karolinska Institute (Diarie number: 2024‐06048‐01).

## Data Availability

The authors have nothing to report.
